# Immortalization effect of SV40T lentiviral vectors on canine corneal epithelial cells

**DOI:** 10.1186/s12917-022-03288-3

**Published:** 2022-05-16

**Authors:** Long Guo, Zhihao Wang, Jun Li, Jianji Li, Luying Cui, Junsheng Dong, Xia Meng, Chen Qian, Heng Wang

**Affiliations:** 1 College of Veterinary Medicine, Jiangsu Co-Innovation Center for Prevention and Control of Important Animal Infectious Diseases and Zoonoses, Yangzhou University, Yangzhou, 225009 Jiangsu China; 2 Joint International Research Laboratory of Agriculture and Agri-Product Safety of the Ministry of Education, Yangzhou, 225009 Jiangsu China

**Keywords:** Canine, Corneal epithelial cell, SV40T, Immortalization, *Staphylococcus pseudintermedius*, Inflammation

## Abstract

**Background:**

Primary canine corneal epithelial cells (CCECs) easily become senescent, and cell proliferation is limited. Therefore, sampling for experimentation requires a large number of animals, which is problematic in terms of animal welfare and fails to maintain the stability of the cells for in vitro analyses.

**Results:**

In this study, CCECs were separated and purified by trypsin and dispase II enzymatic analysis. Next, the cells were immortalized by transfection with a lentiviral vector expressing Simian vacuolating virus 40 large T (SV40T). The immortalized canine corneal epithelial cell line (CCEC-SV40T) was established by serial passages and monoclonal selection. The biological characteristics of CCEC-SV40T cells were evaluated based on the cell proliferation rate, cell cycle pattern, serum dependence, karyotype, and cytokeratin 12 immunofluorescence detection. In addition, we infected CCEC-SV40T cells with *Staphylococcus pseudintermedius* (*S. pseudintermedius*) and detected the inflammatory response of the cells. After the CCEC-SV40T cells were passaged continuously for 40 generations, the cells grew in a cobblestone pattern, which was similar to CCECs. The SV40T gene and cytokeratin 12 can be detected in each generation. CCEC-SV40T cells were observed to have a stronger proliferation capacity than CCECs. CCEC-SV40T cells maintained the same diploid karyotype and serum-dependent ability as CCECs. After CCEC-SV40T cells were infected with *S. pseudintermedius*, the mRNA expression levels of NLRP3, Caspase-1 and proinflammatory cytokines, including IL-1β, IL-6, IL-8 and TNF-α, were upregulated, and the protein levels of MyD88, NLRP3 and the phosphorylation of Iκbα and p65 were upregulated.

**Conclusions:**

In conclusion, the CCEC-SV40T line was successfully established and can be used for in vitro studies, such as research on corneal diseases or drug screening.

**Supplementary Information:**

The online version contains supplementary material available at 10.1186/s12917-022-03288-3.

## Background

Corneal ulcers are caused by trauma, infection, keratoconjunctivitis or eyelid diseases are among the most common and important clinical corneal diseases in dogs [1]. Some corneal ulcers can develop rapidly and threaten the animal’s vision. The pathogenesis of ocular infectious diseases is determined by the virulence of microorganisms, the host defense ability, and the anatomical characteristics of the affected sites [2]. There is no blood supply in the cornea, and this structure is easily damaged by mechanical or biological stimuli. In particular, when the cornea is injured and pathogenic microorganisms invade the structure, increased damage is observed. Many bacteria have been reported to induce corneal infection. For example, *Staphylococcus aureus*, *Streptococcus pneumoniae*, and *Pseudomonas aeruginosa* have significantly higher adhesion rates to ulcerated corneal epithelium than other bacteria, which may be part of the reason why they are often isolated in ulcerative keratitis [3].

Corneal epithelial cells cover the surface of the cornea and act as a barrier against microbial invasion. The average lifespan of corneal epithelial cells is 7–10 days [4], and the stem cells located at the corneal limbus are constantly renewing corneal epithelial cells [5]. Corneal epithelial stem cells account for less than 10% of the total limbal epithelial cells and produce proliferative daughter cells, which are called transient amplifying cells [6]. Abundant evidence has shown that corneal epithelial cells proliferate in the form of centripetal movement from the periphery to the center during the process of physiological proliferation and pathological repair [7]. After denaturation, apoptosis and exfoliation of the central corneal epithelium, the transient amplifying cells migrate from the limbus to the center to replace the terminally differentiated cells. In vitro, corneal epithelial cells are commonly used to study cellular metabolites, pathogenic infections, drug screening and various growth factors affecting cell growth. CCECs easily become senescent in vitro, and supplying sufficient cells for in vitro experiments is difficult, resulting in poor repeatability. A large number of experimental animals are required for scientific research [8]. Although corneal epithelial cell lines of different animals have been cultured in vitro, such as humans [9–11], rats [12] and rabbits [13], the data generated using these cell lines for canine corneal research were not specific. Therefore, an immortalized canine corneal epithelial cell line was established and passaged continuously in vitro, and it has the same biological characteristics as primary corneal epithelial cells.

There are two main methods of establishing cell lines. The first is through the expression of viral oncogenes, such as the adenovirus E1A/E1B gene, simian vacuolating virus 40 large T antigen gene, human papilloma virus E6/E7 gene, etc. The second is through the expression of the Human telomerase reverse transcriptase (hTERT) gene. Studies have shown that when the SV40 large T antigen (SV40T) gene introduced into cells, it can immortalize cells by circumventing the cell cycle M1 through inhibiting the p53 and p16 pathways [14–16].

In this study, we isolated and purified CCECs from canine corneal tissues. A cell line was established through a lentiviral vector containing the SV40T gene. The biological characteristics were evaluated and compared with those of CCECs. The results showed that CCEC-SV40T retained the key characteristics of CCECs and thus could provide a stable cell line for canine corneal research.

## Results

### Isolation and culture of CCECs

**Fig. 1:**
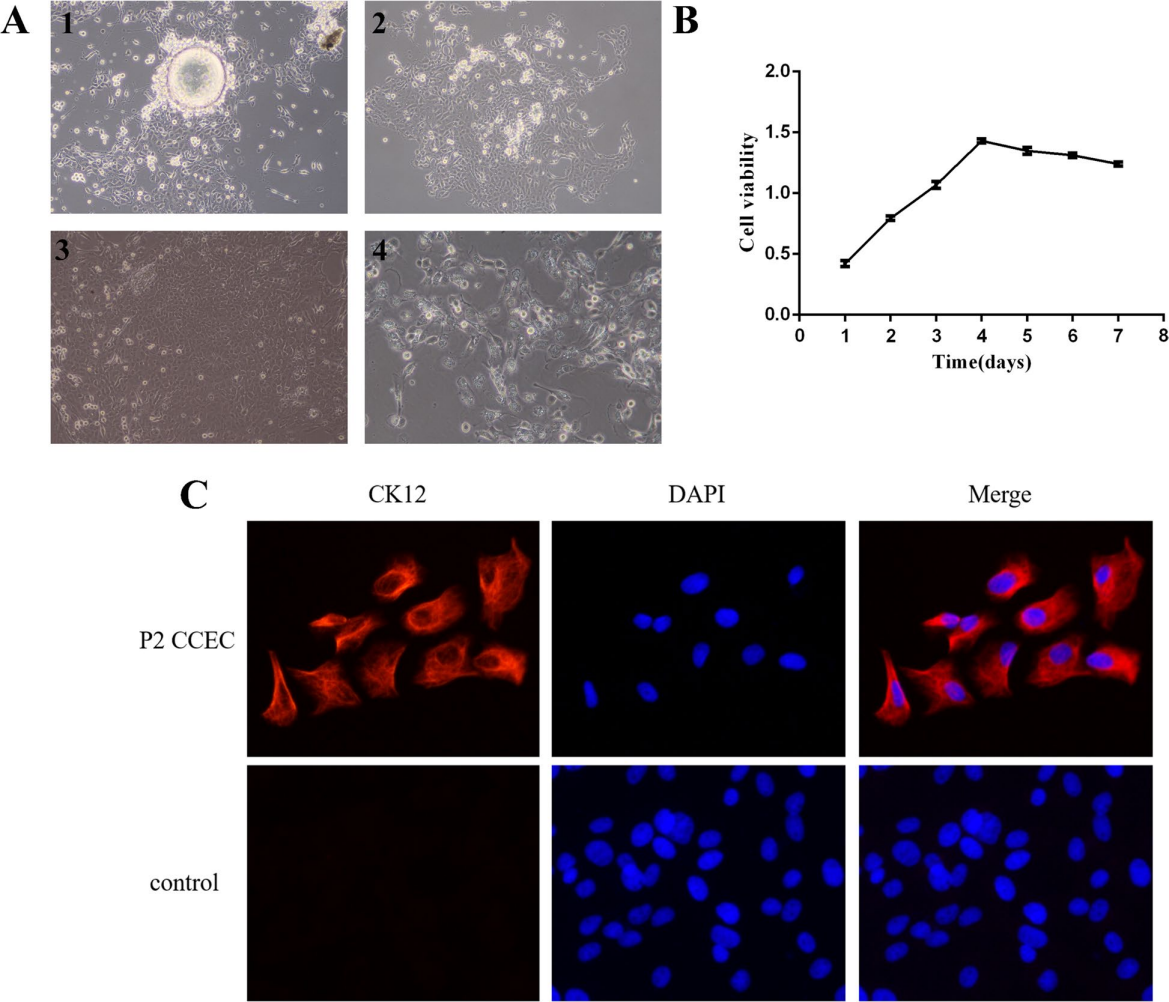
Characteristics of primary canine corneal epithelial cells (CCECs). **A** CCECs observed under a light microscope (1–3 100 × ; 4 200 ×). **B** Growth curve of CCECs at 1 ~ 7 days of culture. **C** Immunofluorescence assays of CCECs for cytokeratin 12

In the process of digestion, a small number of epithelial cells were released as single cells while most were in the form of cell clumps. After the cells and cell clumps were seeded in the cell flask, epithelial cells began to migrate out of the cell clumps on the 2nd day (Fig. 1 A1). On the 5th day, most of the cell clumps showed epithelial cell migration and the cells looked like paving stones under the microscope (Fig. 1 A2). Some clumps desquamated after the cells migrated. After removing the tissue clumps, the epithelial cells grew to confluence on days 7–8 (Fig. 1 A3). The cells were usually aged from 5 to 6 generations (Fig. 1 A4).

The cell proliferation capacity was evaluated by a cell growth curve. The CCECs showed a growth pattern similar to that of normal mammalian cells. (Fig. 1B). The cell immunofluorescence results suggested that cytokeratin 12 was positively expressed in all cells (Fig. 1C).

### Establishment of an immortalized canine corneal epithelial cell line (CCEC-SV40T)

**Fig. 2:**
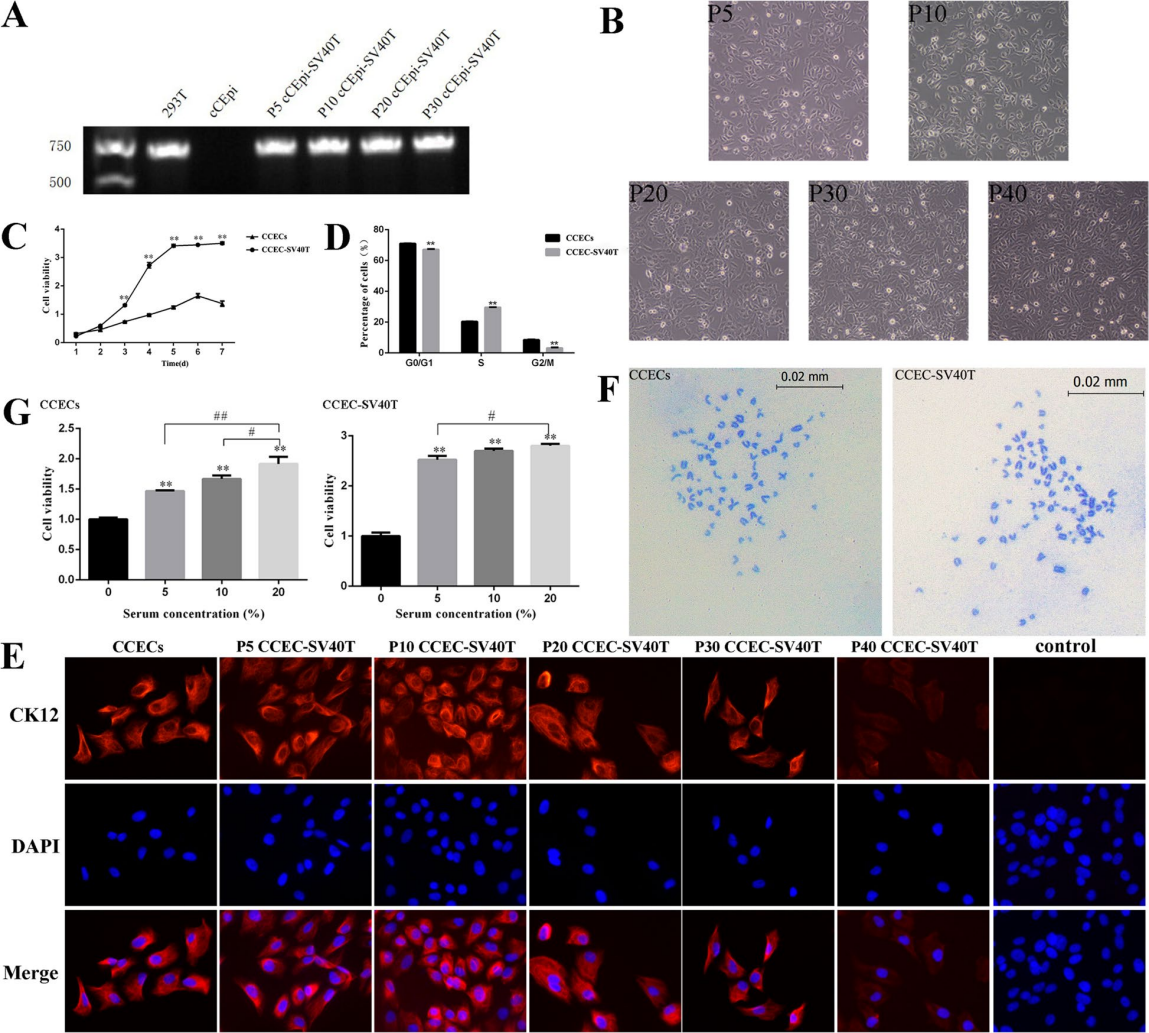
Characteristics of the CCEC-SV40T line. **A** PCR assays of SV40T mRNA. SV40T was detected in 293 T cells and different generations of CCEC-SV40T cells but not in CCECs. **B** Cellular morphology of CCEC-SV40T cells at 5, 10, 20, 30 and 40 generations. No morphological differences were observed. **C** Comparison of the proliferation ability between CCEC-SV40T cells and CCECs. The proliferation rate of CCEC-SV40T cells increased significantly compared with that of CCECs after 3 days (**P < 0.01). **D** Comparison of the cell cycle between CCECs and CCEC-SV40T cells. The percentage of cells in S phase was significantly higher for CCEC-SV40T than for CCEC-SV40T cells (**P < 0.01). **E** Immunofluorescence assays of different generations of CCEC-SV40T for cytokeratin 12. **F** Karyotype analysis of CCEC-SV40T cells and CCECs. **G** Serum dependence analysis of CCEC-SV40T cells and CCECs. Compared with 0%, 5%, and 10% serum concentrations, 20% serum concentrations significantly promoted cell proliferation (**P < 0.01 vs 0% group; #*P* < 0.05, ##*P* < 0.01 vs 20% group)

In this study, the SV40T gene was used to immortalize canine corneal epithelial cells. The PCR results showed that the SV40T gene was not expressed in CCECs but could be detected in 293 T cells and different generations of CCEC-SV40T cells (Fig. 2A). These results showed that the SV40T gene was successfully transfected into canine corneal epithelial cells and stably expressed with the passage of cells.

### CCEC-SV40T cells retain the characteristics of CCECs

During passage, the CCEC-SV40T cells did not change in morphology (Fig. 2B). The proliferation characteristics of the CCEC-SV40T cells were evaluated by cell growth curves and the cell cycle. The proliferation rate of CCEC-SV40T cells was significantly faster than that of CCECs (*P* < 0.05; Fig. 2C). Cell cycle tests showed that the proportion of cells in the S phase of CCEC-SV40T cells was larger than that of CCECs (*P* < 0.05; Fig. 2D). The cell immunofluorescence results suggested that the CCEC-SV40T cells were expressed cytokeratin 12 in different generations (red fluorescence; Fig. 2E). A karyotype analysis of the CCEC-SV40T cells was performed. Both the CCEC and CCEC-SV40T cells maintained diploid karyotypes without significant chromosome abnormalities (2n = 78; Fig. 2F).

Cancerous cells lose their serum dependence during infinite passage in vitro. To identify the noncancer characteristics of CCEC-SV40T cells, the cells were treated with different serum concentrations. When the serum concentration was 0%, the CCECs in the second generation and the CCEC-SV40T cells in the 30th generation could not grow normally. Serum concentrations in the culture medium of 5%, 10%, and 20% all promoted the growth of cells. In addition, 20% serum had a more pronounced proliferation effect than 5% serum (*P* < 0.05; Fig. 2G). The dependence of CCEC-SV40T cells on serum did not change during passage, and CCEC-SV40T cells did not undergo carcinogenesis.

### *S. pseudintermedius* can activate CCEC-SV40T inflammation

**Fig. 3:**
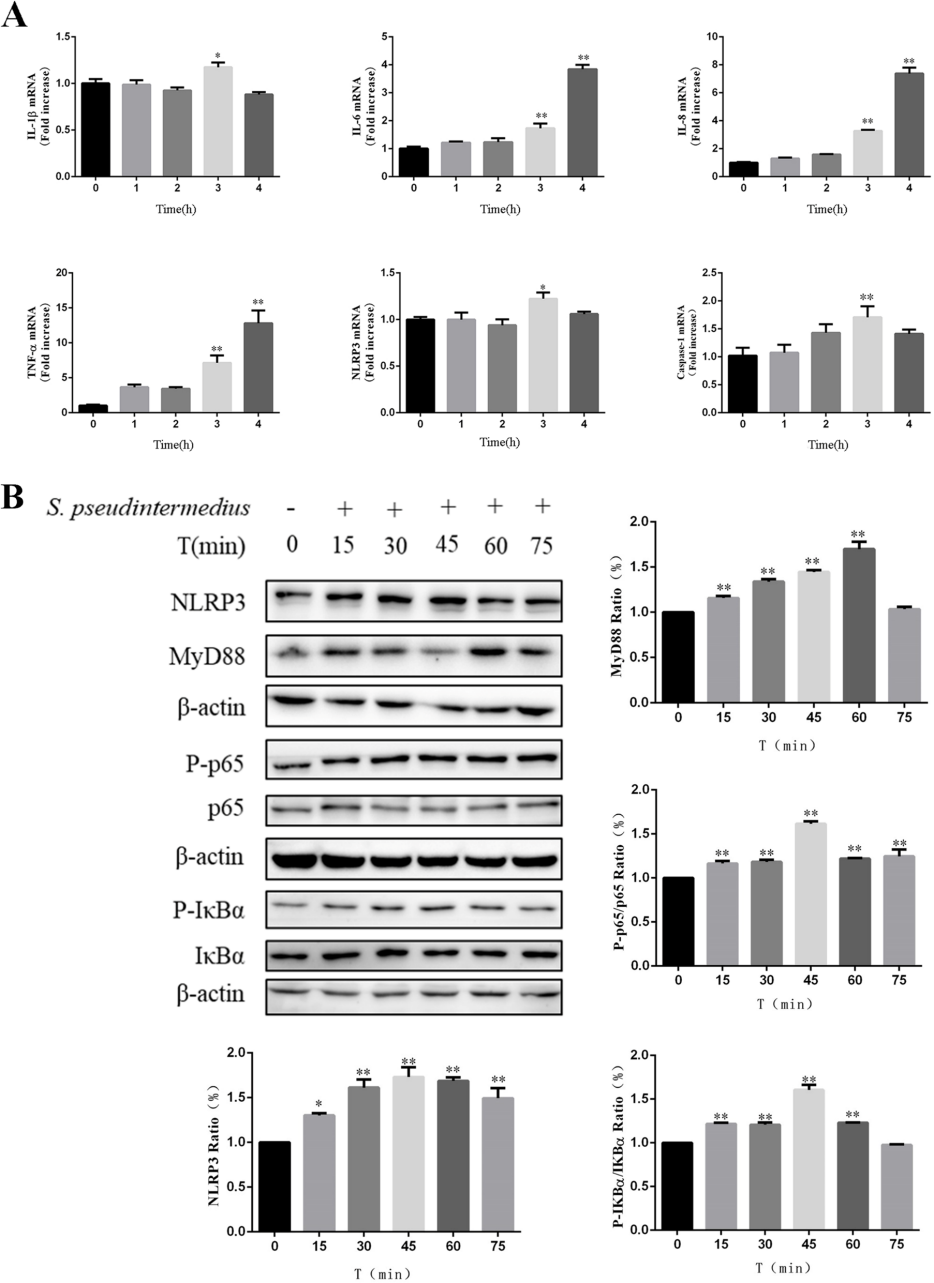
Inflammation of the CCEC-SV40T activated by *S. pseudintermedius*. **A** Expression of proinflammatory cytokines in CCEC-SV40T cells after *S. pseudintermedius* infection. (*P < 0.05, **P < 0.01 vs the control group). **B** Effects of *S. pseudintermedius* on key proteins of the NF-κB pathway and NLRP3 inflammasome in CCEC-SV40T cells at different time points (*P < 0.05, **P < 0.01 vs the control group)

IL-1β, IL-6, IL-8, TNF-α, NLRP3 and Caspase-1 mRNA expression are shown in Fig. 3A. The expression of IL-6, IL-8 and TNF-α increased at 3 and 4 h after the cells were infected with *S. pseudintermedius* (*P* < 0.01). However, the expression of IL-1β, NLRP3 and Caspase-1 increased only at 3 h (*P* < 0.01).

Western blotting was performed to detect the effects of CCEC-SV40T on the NLRP3 inflammasome and NF-κB pathways after the cells were infected with *S. pseudintermedius*. As shown in Fig. 3B, at different times (15, 30, 45 and 60 min) after infection, the protein levels of MyD88 and NLRP3 and the phosphorylation of Iκbα and p65 were significantly upregulated compared with those at 0 min (*P* < 0.01).

## Discussion

In this study, we successfully established an immortalized canine corneal epithelial cell line and continuously passaged it to 40 generations in vitro without any signs of senescence. Moreover, the cell line maintained the same biological characteristics as the primary cells.

CCECs are obtained in vitro mainly by tissue block culture and enzyme digestion culture. The advantage of tissue block culture is that it can obtain primary cells with strong proliferation ability; however, only a limited number of cells is obtained and stromal cell contamination occurs. High-purity epithelial cells can be obtained in a short time by enzyme digestion culture; however, after several digestions, the laminin on the cell membrane is partially destroyed, resulting in a longer time for the cell to adhere to the cell culture flask or the cell exfoliated easily. Feeder layer cells or amniotic membrane carriers are often needed to culture primary corneal epithelial cells. Corneal epithelial cells have been successfully isolated from humans [17], rabbits [18], mice [19], rats, horses [20], etc. Studies have successfully isolated canine corneal epithelial cells and cultured them on amniotic membrane or feeder layer cells [1, 21], which is favorable for transplantation but not suitable for extensive in vitro experiments. In another report, a corneal epithelial cell line was obtained by continuous passage of limbal epithelial cells [22], which could be continuously passaged in vitro independently of the amniotic membrane or feeder cells. In our study, epithelial cells filled with the cell flask could be obtained in approximately one week. A corneal epithelial cell-specific cytokeratin 12 assay was performed on the obtained primary cells. The results showed that cytokeratin 12 was expressed in CCECs, which proved that the cells were derived from corneal epithelium and not from conjunctival epithelium or corneal stroma. The use of the dispase II enzyme during the experiment was able to separate the epithelial layer from the stromal layer, thus avoiding contamination with stromal cells. In addition, the epithelial tissue was not digested into single cells with trypsin, which reduced the damage to the cells and facilitated cell adherence to the wall. However, after 5–6 passages in vitro, the cells appeared senescent.

Immortalization is the ability of primary cells to achieve unlimited proliferation as a result of viral infection, exogenous viral gene insertion, radiation, or drug action. Integrating the SV40T gene into the cell genome is a common method of immortalizing cells [23]. The two signaling pathways of p53 and pRb are related to cell senescence and proliferation. By blocking the p53 and pRb signaling pathways, large T antigens can drive quiescent cells to re-enter S phase and escape apoptosis [24]. The final result is to re-enter the cell cycle and make the cells immortal. In this study, the cell cycle results showed that the proportion of CCEC-SV40T cells in S phase was larger than that of CCECs. At present, immortal cell lines of a variety of animal cells have been established by integrating the SV40T gene, such as mouse embryonic fibroblasts [25], mouse astrocytes [26], mouse small intestinal epithelial cells [27], cow rumen epithelial cells [28], and goat mammary epithelial cells [29]. Therefore, we immortalized CCECs by SV40T. The results showed that after introducing SV40T, it could be continuously and stably expressed in cells. The chromosome karyotype of the immortalized cells may change during passage. Previous studies reported that the NP69-LMP1 sub-cell line had a disturbed karyotype after 50 generations [30]. Our study showed that CCEC-SV40T cells maintained a diploid karyotype without significant chromosome abnormalities and remained serum dependent, indicating that CCEC-SV40T was not cancerous. Therefore, in subsequent experiments, we will select cells for no more than 40 generations.

The surface of the eye is nutrient-rich, moist and in direct contact with the outside environment, and many microorganisms make up the microbiome of the eye [31]. In most cases, the ocular microflora remains relatively stable and plays a role in maintaining normal eye health [32]. When body resistance is reduced or the cornea is damaged, opportunistic pathogens can break down corneal epithelial cell defenses and invade the cornea, ultimately causing infection [33]. Studies have demonstrated that infection of immortalized human corneal epithelial cell lines with live *Staphylococcus aureus* or the cell wall components lipoteichoic acid (LTA) or peptidoglycan (PGN) resulted in elevated expression of IL-6, IL-8, and TNF-α mRNA and activation of the NF-κB and MAPK signaling pathways to initiate the innate immune response [34]. Sanhita Roy et al. [35] used *Corynebacterium pseudodiphtheriticum* to infect human corneal epithelial cell lines. qPCR detection of corneal ulcers from patients and infected epithelial cell lines revealed that the mRNA expression of Toll-like receptors, IL-1β, IL-6, and IL-8 was elevated. Activation of the NF-κB signaling pathway, MAPK signaling pathway and NLRP3 inflammasome was detected at the same time. In addition, LPS could increase the expression of inflammatory factors in immortalized human corneal epithelial cells and activate the NF-κB signaling pathway [36, 37]. In our study, CCEC-SV40T cells were infected with *S. pseudintermedius,* which was isolated from a dog with keratitis. The experimental results suggest that the expression of IL-1β, IL-6, IL-8, TNF-α, NLRP3, and Caspase-1 mRNA was increased. The Western blot results indicated that the NF-κB signaling pathway and NLRP3 inflammasome of CCEC-SV40T cells were activated after infection with *S. pseudintermedius*. The above results indicate that the CCEC-SV40T cells retained their sensitivity to pathogenic bacteria and could produce an inflammatory response under bacterial stimulation. Thus, this cell line can be used as a research model for related diseases in vitro.

## Conclusion

In summary, we used the SV40T lentiviral vector to establish an immortalized canine corneal epithelial cell line (CCEC-SV40T) and evaluated the bioactivity of this line. CCEC-SV40T cells retained the biological characteristics of the primary cells (CCECs). Bacterial stimulation tests showed that CCEC-SV40T cells represent a robust in vitro model for canine cornea-related disease studies.

## Methods

### Primary canine corneal epithelial cells culture

Two dogs were clinically examined before the experiments were performed. The corneal epithelial layer and partial stromal layer were collected and then placed in Dulbecco's Modified Eagle Medium/Nutrient Mixture F-12(DMEM-F12) medium. The tissue was rinsed with PBS containing 50 U/mL penicillin and streptomycin, cut into 2 × 2 mm pieces and incubated with 1.2 IU/mL dispase II at 37 °C for 45 min to separate the epithelium layer from the stromal layer. Then, the epithelial tissue was collected, rinsed with PBS three times, and centrifuged at 200 g for 3 min to discard the PBS. The epithelial tissues were cut aseptically, resuspended in DMEM-F12 medium (containing 15% FBS, 4 mmol/L glutamine, 15 ng/mL EGF), seeded in a cell culture flask, and then cultured for 24 h before changing the medium. The medium was changed every two days, and the cells grew to confluence in approximately 1 week.

### Cell culture

The isolated CCECs were seeded in a cell culture flask at a density of 1 × 10^4^ cells/cm^2^ and cultured in a cell incubator at 37 °C containing 5% CO_2_. DMEM-Fl2 (containing 15% FBS, 4 mmol/L glutamine, and 15 ng/mL EGF) medium was changed every 1–2 days. When passaging, the medium was discarded and 0.25% trypsin combined with 0.02% EDTA was added to the flask, and then the medium was cultured at 37 °C and 5% CO_2_ for 3 min until the cells were separated. Then, the cells were collected, rinsed with medium 3 times and seeded in a new cell culture flask at a density of 1 × 10^4^ cells/cm^2^.

### Canine corneal epithelial cell immortalization and monoclonal selection

CCECs were infected overnight with lentivirus expressing SV40 large T-antigen, and 4 μg/mL polybrene was added to improve transfection efficiency. Then, the virus supernatant was discarded and rinsed 3 times with PBS, and fresh medium was added for further culture. After 7 days of culture, the cells were digested by 0.25% trypsin mixed with 0.02% EDTA and collected. The cells were diluted to 5 cells/mL in DMEM-F12 medium, and then 200 μL of cell suspension was added to each well of a 96-well plate, which was observed every 5 days. If cells reached 50% confluence with epithelial morphology, the cells were transferred into a 24-well plate for further culture. Thus, immortalized CCEC-SV40T cells were successfully established.

### Cell proliferation ability analysis

A Cell Counting Kit-8 (CCK-8,Dojindo Molecular Technologies, Japan) was used for the cell proliferation assays according to the manufacturer's protocol. The cells were seeded into a 96-well plate at a density of 5 × 10^2^ cells/well and cultured continuously for 7 days. The cells in 6 wells were collected every day, and the activity of cell proliferation was determined. An automicroplate reader was used to measure the absorbance of each well at a wavelength of 450 nm.

### RT-PCR detection of SV40T transcription

CCEC-SV40T, CCECs and 293 T cells were seeded in 35 mm dishes and cultured for 48 h. According to the manufacturer's protocol, total RNA was extracted using TRIzol reagent. The PrimeScript™ RT kit (Perfect Real Time, TaKaRa) with gDNA eraser was used to convert RNA to cDNA. 2 × EasyTaq PCR SuperMix (Transgen Biotech) and a Bio-Rad PCR detection system were used for real-time PCR. PCR was performed using the following procedure: 1 cycle of denaturation at 94 °C for 300 s; 34 cycles of denaturation at 94 °C for 30 s, annealing at 60 °C for 30 s and 72 °C for an additional 30 s; and 1 cycle at 72 °C for 300 s [38]. The primers are listed in Table 1.

**Table 1:** Primers for SV40T

Gene	Sequences (5’ → 3’)	Product size	References
SV40T	F: AGTGGCTGGGCTGTTCTTTT	671 bp	Zhang Kang et al., 2019 [38]
	R: ATGGGAGCAGTGGTGGAATG		

### Cell immunofluorescence staining

CCECs in the 2nd generation and CCEC-SV40T cells in the 5th, 10th, 20th, 30th, and 40th generations were grown on cover slips in 24-well cell culture plates at a density of 1 × 10^5^ cells/mL. When the cells grew to 70% confluence, they were fixed with 4% paraformaldehyde at room temperature for 15 min. After the cells were washed with PBS 3 times, 0.1% Triton X-100 was used to permeate the cell membranes for 10 min. Then, the cells were blocked with 5% bovine serum albumin at room temperature for 1 h. The cells were incubated with anti-cytokeratin 12 antibody (1:400; Abcam) at room temperature for 1 h and then cocultured with FITC-conjugated goat anti-rabbit IgG antibodies (1:500; Thermo Fisher Scientific) at room temperature for 1 h. The nuclei were stained with DAPI. A confocal laser scanning microscope was used for observation.

### Cell cycle analysis

CCECs in the 2nd generation and CCEC-SV40T in the 30th generation were cultured in 60 mm dishes at a density of 1 × 10^4^ cells/cm^2^. When the cells grew to more than 80% confluence, the cells were collected. The cells were washed with cold PBS 3 times, resuspended in cold 70% ethyl alcohol and fixed at 4 °C for 12 h. The fixed cell suspension was centrifuged at 800 g for 5 min, and the supernatant was discarded. Then, the cells were washed with cold PBS twice and incubated with propidium iodide. The cell cycle was determined by flow cytometry.

### Karyotype analysis

To determine whether the chromosomes of the CCEC-SV40T cells were altered, CCECs in the 2nd generation and CCEC-SV40T cells in the 40th generation were selected for karyotype analysis. The cells were cultured in 6-well plates and treated with 0.1 μg/mL colchicine for 2 h. Then, the cells were centrifuged and incubated with 0.075 mol/L KCl at 37 ℃ for 30 min. The cells were fixed with 2 mL fixative (methanol:glacial acetic acid = 3:1 (v/v)) for 5 min and then centrifuged at 200 g for 5 min to remove the supernatant. Fixative was added to resuspend the cells, and then the cell suspension was placed on a prefrozen glass slide, stained with Giemsa for 10 min, and then air-dried at room temperature. The number of chromosomes was counted under a light microscope.

### Serum dependence analysis

The 2nd generation CCECs and the 30th generation CCEC-SV40T cells were cultured in 96-well plates at a density of 2 × 10^3^ cells/well. When the cells were completely adherent, DMEM-F12 medium containing 0%, 5%, 10% and 20% fetal bovine serum was used for future culture. Three replicate wells were set for each concentration, and the medium containing different concentrations of serum was considered the zero adjustment well. After the cells were cultured for 24 h, the medium was discarded and the cells were washed with PBS 3 times. Then, 100 μL fresh medium (without FBS) and 10 μL CCK-8 reagent were added to each well and incubated for 2 h at 37 °C and 5% CO_2_. An auto microplate reader was used to measure the absorbance of each well at a wavelength of 450 nm.

### Preparation of S. pseudintermedius

*S. pseudintermedius* was incubated in 20 mL liquid Luria–Bertani (LB) culture at 37 °C and 120 r/min. After reaching the logarithmic growth phase, the bacteria were washed with PBS 3 times and diluted with DMEM-F12 medium to achieve a bacterial concentration of infection (MOI = 1:1).

### RNA extraction and qPCR

The CCEC-SV40T cells were treated with *S. pseudintermedius* for 0, 1, 2, 3 and 4 h. Then, the cells were washed with PBS 3 times and the total RNA of the cells was extracted according to the manufacturer's instructions using TRIzol reagent (Thermo, USA). The concentration and purity of the extracted RNA were checked using a NanoDrop 2000 spectrophotometer (Thermo, USA). The ratio of absorption (A260/A280) was between 1.8 and 2.0, and the RNA (900 ng) was reverse transcribed to cDNA with the PrimerScript RT regent Kit gDNA Eraser (Takara, Japan). qPCR was performed using a CFX 96 Real-Time PCR Detection System (Bio-Rad, USA). Amplification mixtures contained 5 μL SYBR Premix Ex Taq™ II (Takara, Japan), 1 μL of each primer, and 1 μL of cDNA template in a final volume of 10 μL per reaction, and the following cycling conditions were performed: 95 ℃ for 2 min; 40 cycles of 95 ℃ for 5 s, 60 ℃ for 30 s; 95 ℃ for 15 s; 60 ℃ for 1 min; and 95 ℃ for 15 s. The 2^−ΔΔCt^ method was used to analyze the relative gene expression (target gene expression normalized to the expression of the endogenous control gene) [39]. The PCR analyses were performed in triplicate. The primer sequences are presented in Table 2.

**Table 2:** qPCR primers used in this study

Gene name	Sequences (5’ → 3’)	Length(bp)	Accession number
IL-1β	F: GGAAATGTGAAGTGCTGCTGCCAA	150 bp	NM_001037971
	R: GCAGGGCTTCTTCAGCTTCTCCAA		
IL-6	F: ACCACTCACCTCTGCAAACA	236 bp	NM_001003301
	R: GCTGAAACTCCACAAGACCG		
IL-8	F: AGGCTGAGAAACAAGATCCGT	128 bp	NM_001003200
	R: ACCAGGTCTACACGGGACAT		
TNF-α	F: GTTGTAGCAAACCCCGAAGC	122 bp	NM_001003244
	R: TACAACCCATCTGACGGCAC		
NLRP3	F: GAGGAGAAGGCATGGGCCATG	154 bp	XM_005623149
	R: CCAATAAACCCAACCACTCCTCTTCAA		
Caspase-1	F: TGGAGCTGAACTTGACATTGCAGG	114 bp	EU183118
	R: AATTCCCGTAGCACTGATTCCATACC		
GAPDH	F: GGGTGATGCTGGTGCTGAGTAT	186 bp	XM_003435649
	R: TTGCTGACAATCTTGAGGGAGTT		

### Western blot analysis

CCEC-SV40T cells were treated with *S. pseudintermedius* for 0, 15, 30, 45, 60 and 75 min. Total protein was extracted and quantified using a bicinchoninic acid protein assay kit (Beyotime, China). Proteins (20–30 μg) were separated by 10% SDS–polyacrylamide gels and then transferred to polyvinylidene difluoride (PVDF, Millipore, Germany) membranes. The PVDF membranes were incubated at room temperature for 1 h in 5% nonfat milk diluted with 0.05% Tween-20 Tris–HCl buffer to block nonspecific binding. The membranes were cropped according to the size of the required protein before incubating with the primary antibodies. The membranes were incubated with primary antibodies specific for β-actin (# 4970), NLRP3 (# 15101S), MyD88 (# 4283), p65 (# 8242), p-p65 (# 3033), IκBα (# 4812), and p-IκBα (# 2859). All primary antibodies were purchased from Cell Signaling Technology, and all were diluted with 5% bovine serum albumin to a 1:1000 dilution at 4 °C overnight. Then, the membranes were incubated with HRP-conjugated secondary antibodies (diluted with 5% nonfat milk to a 1:2000 dilution) at room temperature for 1 h. Proteins were detected using a chemiluminescence (ECL) assay.

### Statistical analysis

All data were analyzed as the mean ± standard error of the mean (SEM). The groups were compared by one-way ANOVA, which was followed by Dunnett’s test (SPSS 18.0 software). *P* < 0.05 indicated a significant difference between groups. Data are shown in column bars representing the mean ± SEM of at least three independent experiments.

## Supplementary Information

**Additional file 1.**

## Data Availability

The datasets analyzed during the current study are available from the corresponding author on reasonable request.

## Abbreviations

CCECs: Primary canine corneal epithelial cells
CCEC-SV40T: Immortalized canine corneal epithelial cell line
CCK-8: Cell Counting Kit 8
DMEM-F12: Dulbecco's Modified Eagle Medium/Nutrient Mixture F-12
EDTA: Disodium Ethylenediamine Tetra Acetate
EGF: Epidermal Growth Factor
FBS: Fetal Bovine Serum
hTERT: Human telomerase reverse transcriptase
IL: Interleukin
LB: Luria–Bertani
LPS: Lipopolysaccharide
LTA: Lipoteichoic acid
MAPK: Mitogen-activated protein kinase
MyD88: Myeloid differentiation factor 88
NF-κB: Nuclear transcription factor kappa B
NLRP3: NLR family pyrin domain containing 3
PBS: Phosphate buffer saline
PGN: Peptidoglycan
PVDF: Polyvinylidene difluoride
qPCR: Real-time quantitative reverse transcription PCR
RT-PCR: Reverse transcription-PCR
*S. pseudintermedius*: *Staphylococcus pseudintermedius*
SV40T: Simian vacuolating virus 40 large T
TNF: Tumor necrosis factor
