# Supplementary material for: Immortalization effect of SV40T lentiviral vectors on canine corneal epithelial cells
Source: BMC Vet Res. 2022 May 16;18:181. doi: 10.1186/s12917-022-03288-3 (PMC9109393; doi:10.1186/s12917-022-03288-3)

PCR gel-SV40T
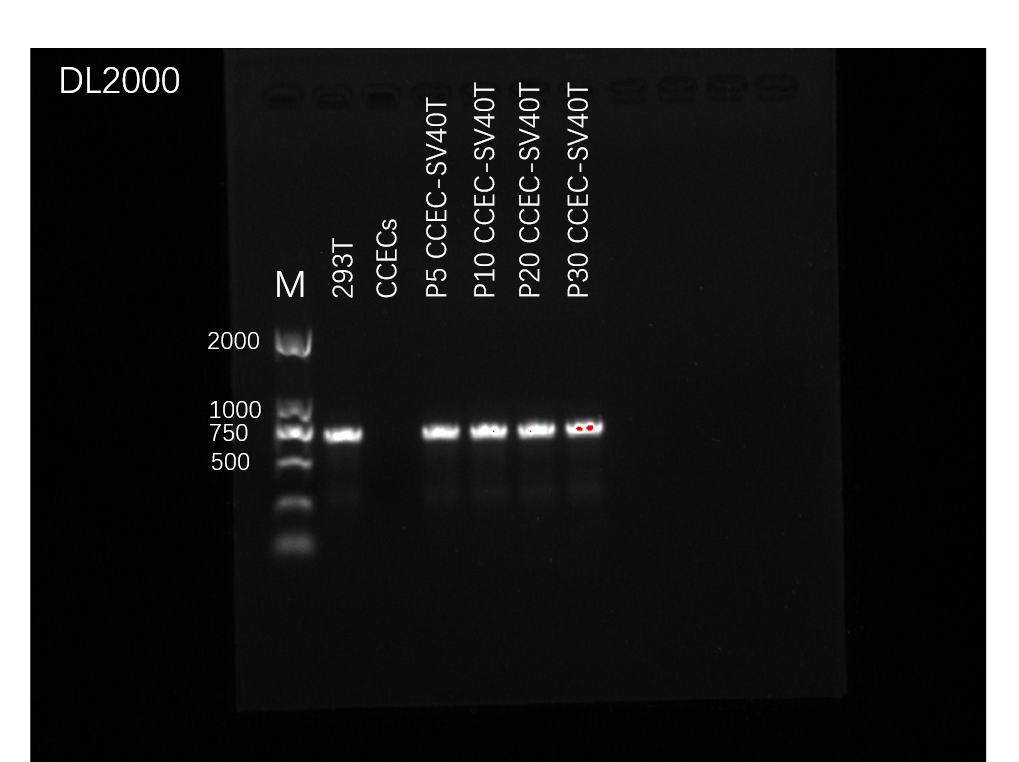


1-IKB


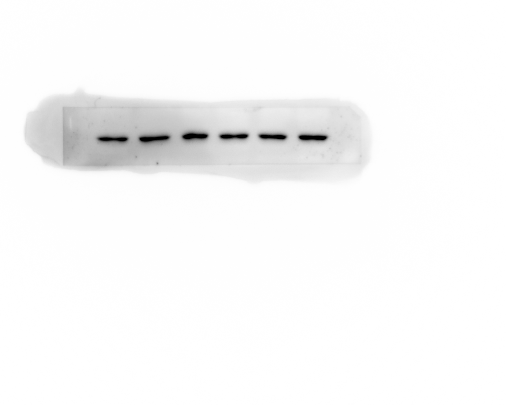


1-P-IKB


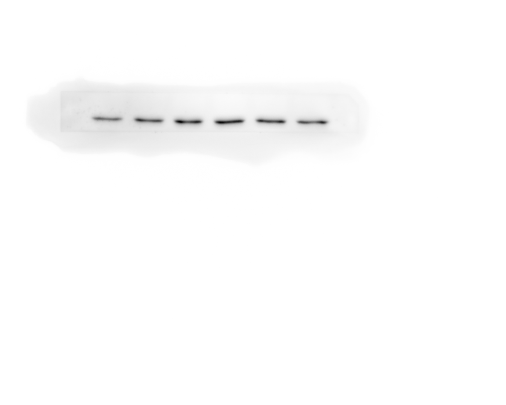


2-IKB


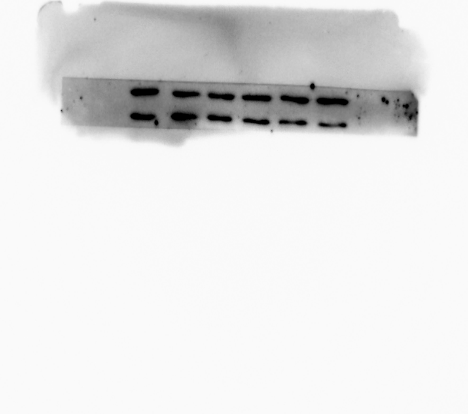


2-P-IKB


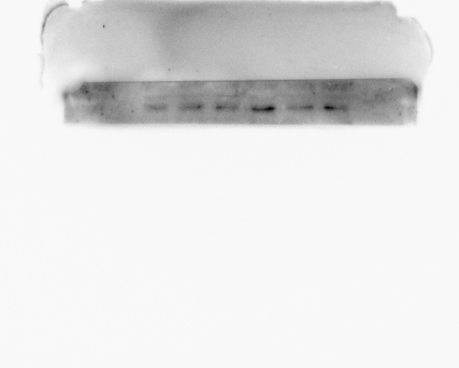


3-IKB


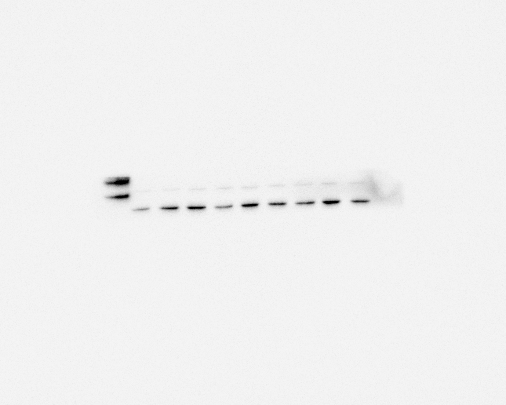


3-P-IKB


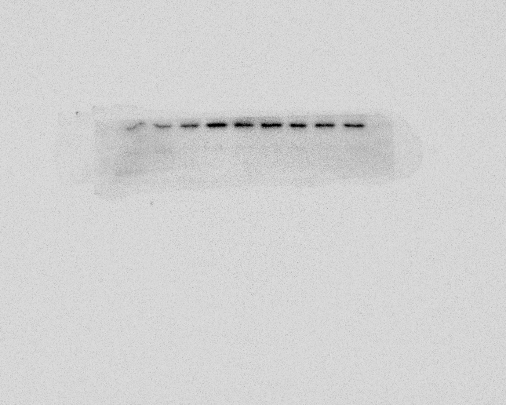


1-P65


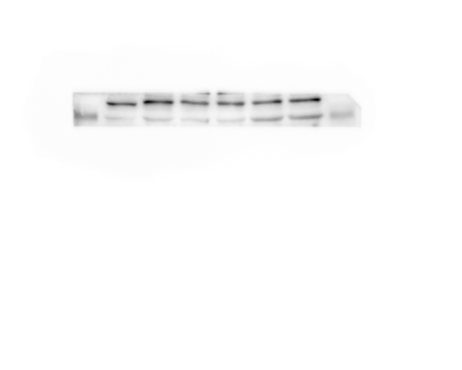


1-P-P65


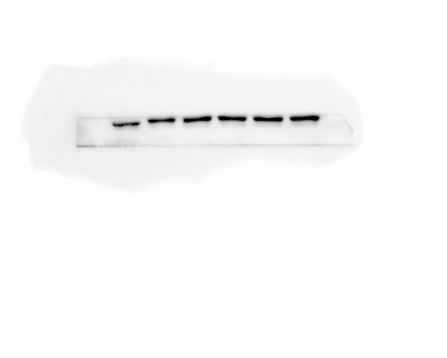


2-P65


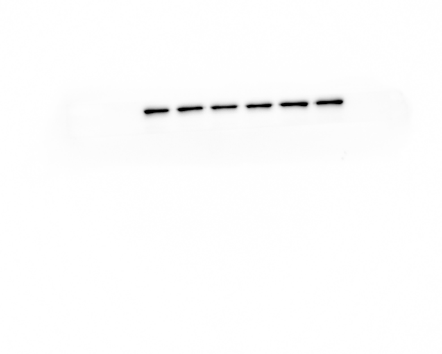


2-P-P65


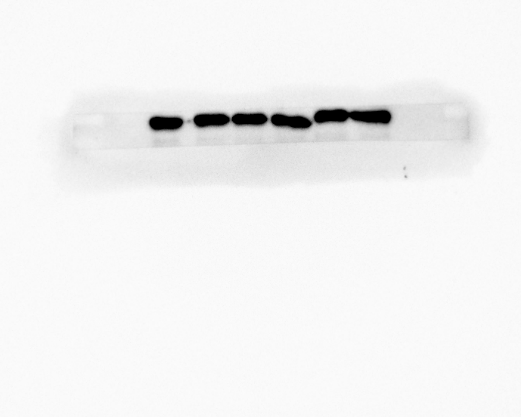


3-P65


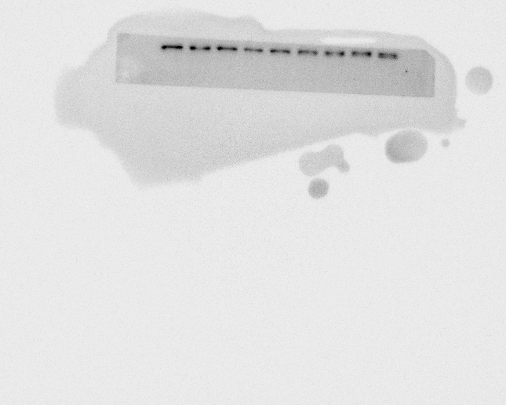


3-P-P65


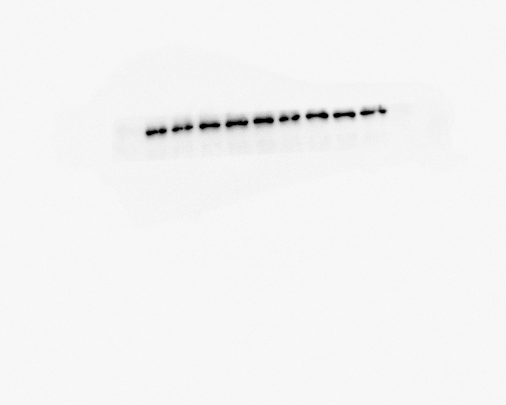


1-MyD88


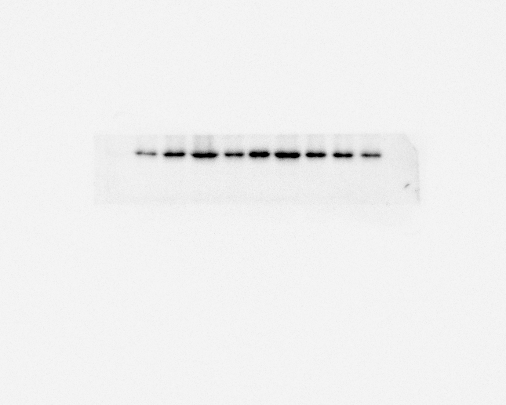


1-β-actin

NLRP3


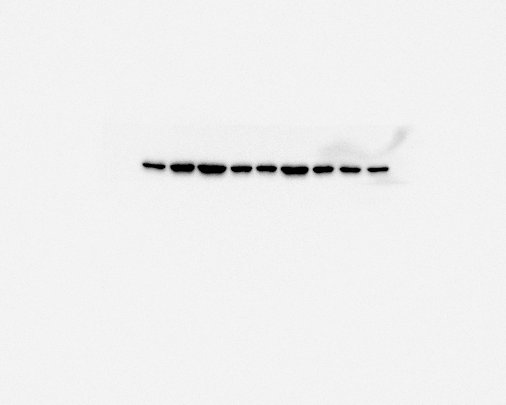


β-actin

2-MyD88


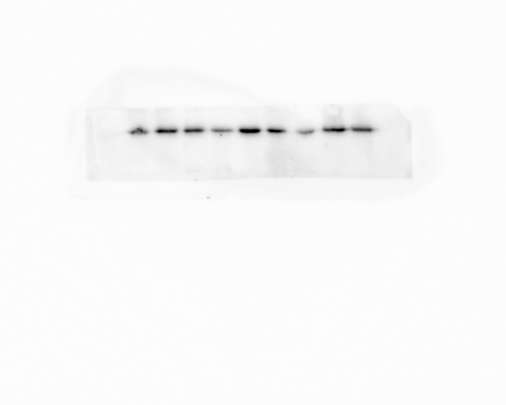


2-β-actin


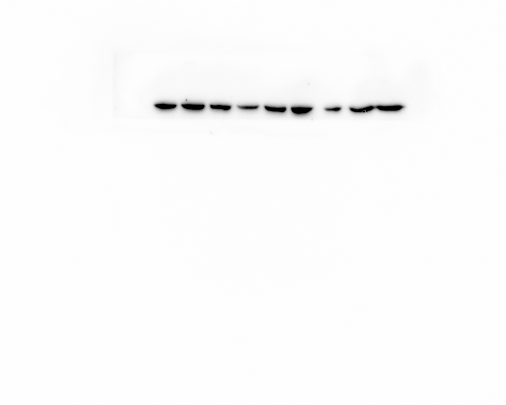


3-MyD88


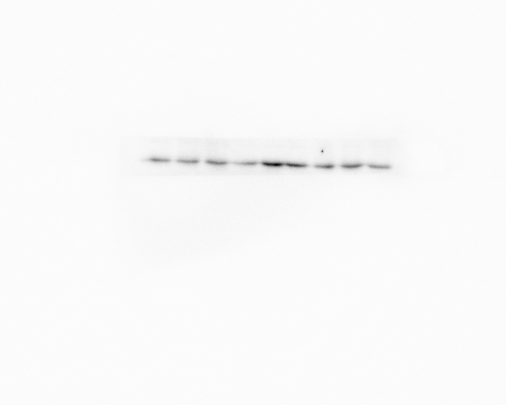


3-β-actin


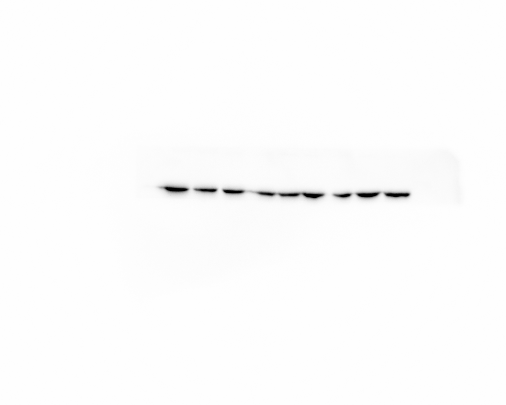


1-NLRP3


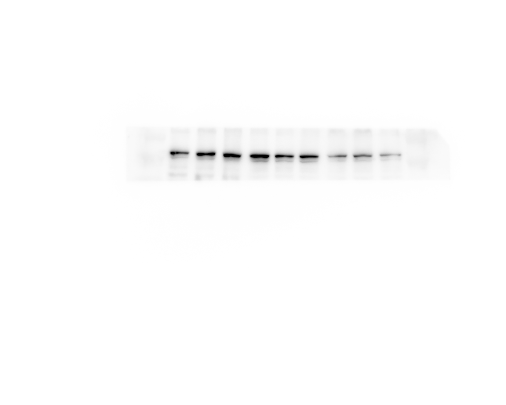


1-β-actin


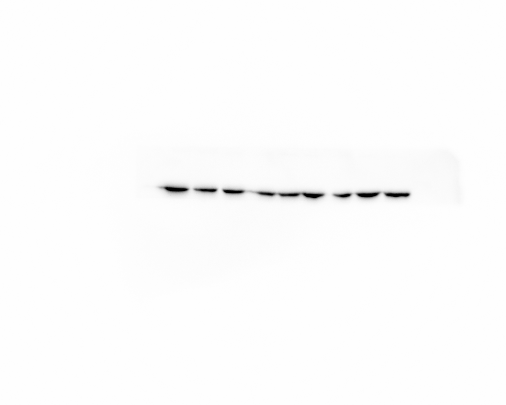


2-NLRP3


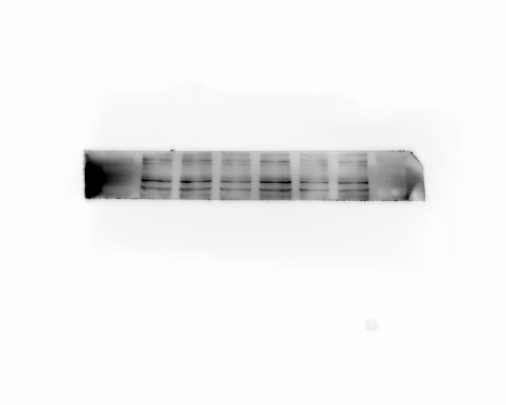


2-β-actin


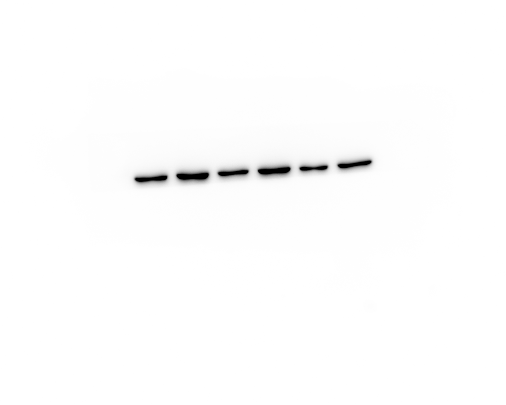


3-NLRP3


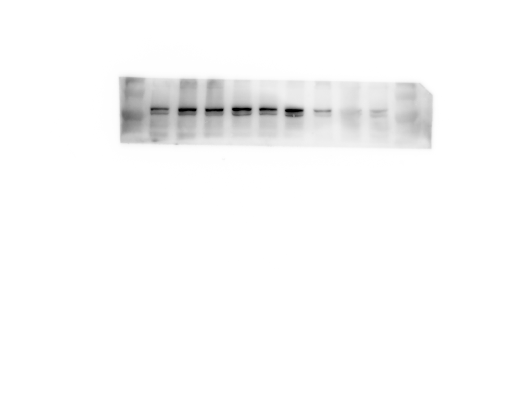


3-β-actin


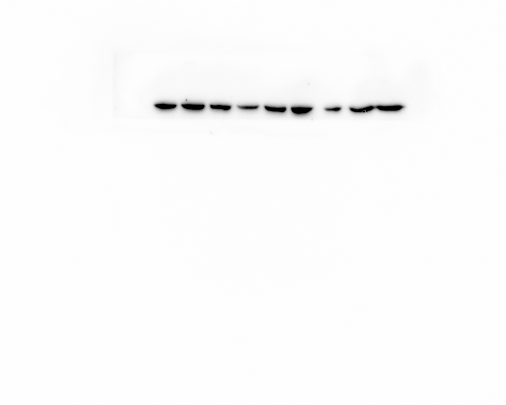

Supplement: Supplementary file 1 — Additional file 1. [file 12917_2022_3288_MOESM1_ESM.docx]
